# Liquid Supercoolability and Synthesis Kinetics of Quinary Refractory High-entropy Alloy

**DOI:** 10.1038/srep37191

**Published:** 2016-11-16

**Authors:** W. L. Wang, L. Hu, S. J. Yang, A. Wang, L. Wang, B. Wei

**Affiliations:** 1 Department of Applied Physics, Northwestern Polytechnical University, Xi’an 710072, P. R. China

## Abstract

The high-entropy configuration of equiatomic multicomponent alloys opens an effective access to the development of advanced materials. Here we report the synthesis of a new quinary refractory WMoTaNbZr high-entropy alloy under electrostatic levitation condition. It showed a high liquidus temperature of 2686 K and achieved a maximum supercooling of 640 K (0.24 *T*_*L*_) at molten state. The containerless measurements revealed a linear increasing tendency for both its liquid state density and the specific heat to emissivity ratio versus alloy supercooling. A high-entropy body-centered cubic (HEB) phase dominated its phase constitution despite the formation of a negligible amount of solid solution (Zr) phase. The dendritic growth of HEB phase always governed the crystallization process, attained a fastest growth velocity of 13.5 m/s and displayed a power function relation to alloy supercooling. The high speed videographic research of recalescence phenomenon indicated Johnson-Mehl-Avrami type transition kinetics for its rapid solidification process. As supercooling increases, the microstructures of primary HEB phase were refined conspicuously and exhibited an obvious solute trapping effect of the segregative Zr component. Meanwhile, the Vickers hardness of HEB phase displayed the rising tendency with supercooling.

High-entropy alloys (HEAs) have aroused great scientific research interest owing to their high strength, good ductility and strong corrosion resistance together with simple solid-solution phase structure^1^,^2^,^3^,^4^,^5^,^6^,^7^. Since the pioneering work of Yeh and coworkers^2^, the CoCrFeNiCu/Mn/Al HEAs have been most extensively investigated because of their satisfactory thermodynamic stability at both liquid and solid states^1^,^2^,^4^,^8^,^9^,^10^. In order to develop advanced materials for ultrahigh temperature applications, it is quite desirable to design and synthesize refractory especially tungsten-involving HEAs. But their extremely high melting temperatures and violent chemical reactivity at molten state bring in great difficulties for the practical synthesis of this category of alloys. So far, there have been a few published studies about the experimental preparation and property analysis of refractory HEAs. Senkov *et al*.^1^,^2^,^5^,^8^,^11^,^12^,^13^ succeeded in synthesizing the refractory WMoTaNb/V HEAs and explored their microstructural evolution and mechanical behavior. Zou and coworkers^14^,^15^ developed an ultrastrong ductile quarternary WMoTaNb alloy. Koželj *et al*.^4^ discovered a superconducting Ta_34_Nb_33_Hf_8_Zr_14_Ti_11_ HEA. Nevertheless, it is still rare to find published reports about the liquid state supercoolability, thermophysical properties, dendrite growth kinetics and mechanical behavior of supercooling for HEAs.

The thermophysical properties such as the density and specific heat of liquid high-entropy alloys, particularly those in supercooled state, are their essential parameters to accomplish the material design, investigate the solidification process kinetics and optimize the microstructure and properties^16^,^17^,^18^,^19^,^20^,^21^,^22^. In the specific case of refractory tungsten-involving HEAs, almost all the conventional container-based measurement techniques become inadequate. The realization of a remarkable degree of liquid supercooling requires for the effective prevention of the heterogeneous nucleation caused by the chemical reactions and container walls. Although the containerless processing by various levitation techniques^15^,^17^,^21^,^23^,^24^,^25^ is a possible way to overcome this technical barrier, the experimental attempt to supercool liquid refractory HEAs and measure their thermophysical properties still remains to be a sort of adventure.

Due to their single phase constitution, the dendrite growth of solid solution phase is the governing pattern formation mechanism during the rapid solidification of supercooled liquid high-entropy alloys^26^,^27^,^28^,^29^. There have been numerous investigations on the microstructure characteristics and their relations to applied properties even for refractory HEAs^30^,^31^,^32^,^33^. Yet it is of more crucial importance to quantitatively measure the dendrite growth velocity versus liquid alloy supercooling for an in-depth study of their synthesis kinetics. Some significant progress has been made for traditional multicomponent alloys in this respect^34^,^35^,^36^,^37^,^38^,^39^, which reveals double exponential growth kinetics and concurrent solute trapping effects of interactive elements. However, HEAs are quite unusual because of their equiatomic composition and no principal component as solvent. In fact, every component behaves as both solvent and solute during the dendritic growth of HEAs. This may induce novel kinetic effects in such aspects as the advancing mechanism of solid-liquid interface and the dynamic profiles of multicomponent redistribution.

Electrostatic levitation (ESL) technique assisted with laser heating method provides an effective approach to synthesize refractory tungsten-involving high-entropy alloys. Meanwhile, high speed CCD videography opens a new access to the dynamic and digital analysis of transient thermal recalescence process. The objective of this work is to design and synthesize a novel refractory high-entropy WMoTaNbZr alloy under ESL condition. Its liquid supercoolability, nucleation characteristics, and thermophysical properties were explored under containerless state. Special attention was paid to the dendritic growth kinetics and the microstructure evolution mechanism of this substantially supercooled refractory high-entropy alloy.

The master alloy with quinary WMoTaNbZr equiatomic composition was prepared by arc melting technique from component metals of high purity better than 99.95%. The electrostatic levitation (ESL) apparatus insured an ultrahigh vacuum level of 10^−5^ Pa and was incorporated with Trumpf HL 1006D YAG heating laser of 1064 nm and 1 kW. Each levitated sample had a mass of 200 mg and a diameter of 3 mm. After superheated to 300 K above its liquidus temperature, it was naturally cooled by switching off the laser power. Its temperature profile and supercooling level were monitored by a DIAS 10 N single-color pyrometer with an absolute accuracy of +5 K and measuring range of 1273~4023 K. The lightening-like recalescence phenomenon was recorded by a Redlake MotionXtra HG-100K high speed CCD camera with a frame rate up to 3 × 10^5^ fps, while an infrared photo diode device was also used to measure the dendritic growth velocity. Every sample was subjected to the melting- solidifying cycle for 3~5 times. Their phase constitutions were analyzed by Rigaku D/max 2500 X-ray diffractometer (XRD), whereas their solidification structures and solute distribution profiles were investigated with FEI Sirion scanning electron microscope (SEM) and INCA 300 energy dispersive spectrometer (EDS), Vickers hardness was measured by Struers Dura-min A300 testing machine with a load of 2.94 N.

## Determined liquidus temperature and supercooling by ESL

**Figure 1:**
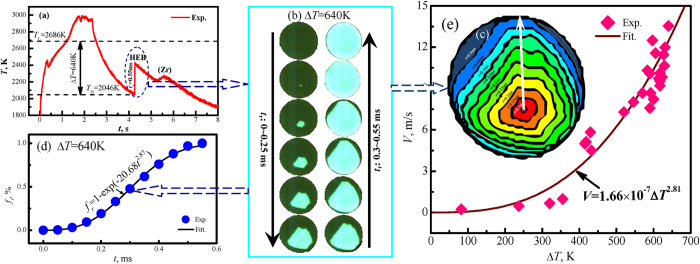
The dynamic supercooling and recalescence processes of liquid refractory WMoTaNbZr high-entropy alloy. (**a**) The measured temperature profiles of heating and cooling curves for a sample supercooled by 640 K, (**b**) a continuous series of high speed CCD images recording the thermal recalescence phenomenon, (**c**) the recalescence volume fraction versus time displaying Johnson-Mehl-Avrami type transition kinetics, (**d**) the synthesized schematics of the advancing solidification front, and (**e**) the measured dendritic growth velocity as a power function of liquid alloy supercooling.

The refractory WMoTaNbZr high-entropy alloy was successfully synthesized by the containerless rapid solidification under electrostatic levitation condition (ESL). Due to the liquidus temperature of refractory HEAs cannot be measured by traditional thermal analysis techniques, we calibrated the temperature profile of the infrared pyrometer by the high accuracy WRe3-WRe25 thermocouple. The liquidus temperature of WTaMoNbZr alloy was determined as 2686 K with an error ±10 K after multiple repeated calibrations. The calibrated temperature profile of heating and cooling curves for a sample is shown in Fig. 1(a). During the solidification, the thermal recalescence phenomenon always occurs, and the inflection point of the first recalescence peak determines the supercooling value. The supercooling Δ*T* is defined as the temperature difference between the liquidus temperature *T*_*L*_ and the nucleation temperature *T*_*N*_ of the primary phase, that is Δ*T* = *T*_*L*_−*T*_*N*_. As illustrated in Fig. 1(a), the maximum supercooling of 640 K (0.24 *T*_*L*_) is achieved in the experiments under the containerless condition. The recalescence process lasts for 0.55 ms. In the process of the rapid solidification of highly supercooled liquid alloy, the nucleation point is generally distributed on the surface of levitated melts (Fig. 1(b,c)). The solidification front rapidly migrates in the racalescence stage. After that, the sample is composed of the primary dendrites and residual liquid phase. Fig. 1(d) presents the solidified volume fraction during recalescence (*f*_*r*_). It is clear that the volume fraction increases slowly at the beginning of nucleation where the recalescence time *t*_*r*_ is lower than 0.1 ms. However, it increases rapidly with recalescence time, *f*_*r*_ = 88.1% at *t*_*r*_ = 0.45 ms. Finally, *f*_*r*_ = 100% at *t*_*r*_ = 0.55 ms. The volume fraction of recalescence characteristic (*f*_*r*_) is demonstrated as the Johnson-Mehl-Avrami equation:


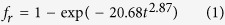


## Thermophysical properties of refractory WMoTaNbZr HEAs

**Figure 2:**
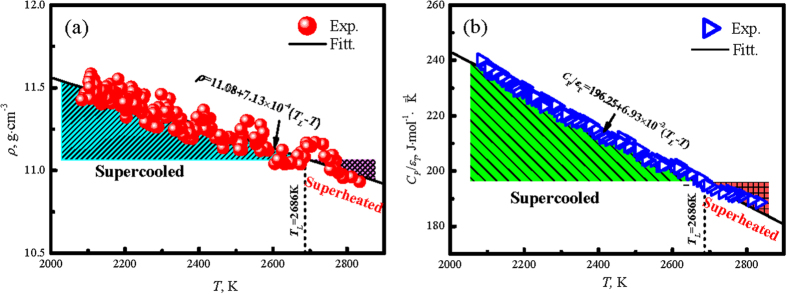
The thermophysical properties of liquid refractory WMoTaNbZr high-entropy alloy versus temperature measured under electrostatic levitation condition, (**a**) the density variation at both superheated and supercooled liquid states, and (**b**) the ratio *C*_*P*_/*ε*_*T*_ of specific heat to hemispherical emissivity increases linearly with supercooling.

Under the electrostatic levitation condition, the sample is levitated in a high vacuum environment, and it will naturally cool down when the laser is turned off. The density and the specific heat to emissivity ratio of the refractory WMoTaNbZr high-entropy alloy can be acquired during the experiment. From Fig. 2(a), the density is measured according to *ρ* = *m*/*V, m* is the sample mass, and *V* is the sample volume. The density of superheated and superercooled liquid alloy exhibits a liner increase with the supercoiling (Δ*T* = *T*_*L*_−*T*) and can be fitted by the following expression:


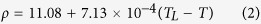


Meanwhile, the specific heat to emissivity ratio for the refractory WMoTaNbZr high-entropy alloy is determined by the following equation:


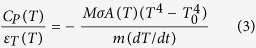


where *C*_*P*_(*T*)/*ε*_*T*_(*T*) is the ratio of isobaric heat capacity to hemispherical total emissivity, *M* is the molar mass, *σ* is the Stefan-Boltmann constant, *A*(*T*) is the sample surface area, *T*_0_ is the surrounding temperature, *t* is the time, and d*T*/d*t* is the cooling rate which can be obtained from cooling curves. The results present the similar tendency to density, that is the smaller value of *C*_*P*_(*T*)/*ε*_*T*_(*T*) in the superheated zone, and it increases with the decrease in temperature, which is illustrated in Fig. 2(b), the larger supercooling, the higher *C*_*P*_(*T*)/*ε*_*T*_(*T*), which exhibits a linear rise with the decrease of the temperature and increase of the supercoiling and can be described by:


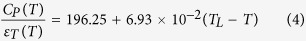


## Dendritic growth kinetics mechanism under high supercooling

**Figure 3:**
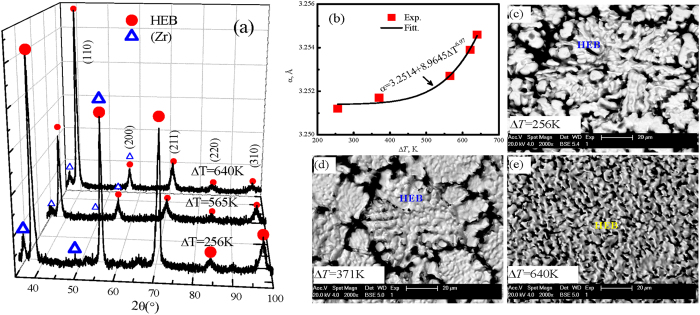
The phase constitution, lattice constant and surface microstructure of rapidly solidified refractory WMoTaNbZr high-entropy alloy with different supercoolings, (**a**) the XRD patterns of three samples supercooled by 256, 565 and 640 K respectively, (**b**) the lattice constant of HEB phase increases with liquid alloy supercooling, and (**c**–**e**) the BSE images for the original free surfaces of three containerlessly solidified samples.

Figure 3(a) shows the X-ray diffraction patterns of refractory WMoTaNbZr high-entropy alloy. The crystal structure of these alloys includes two phases at the different supercoolings: the major high-entropy body-centered cubic (bcc) phase and the minor solid solution (Zr) phase. The high-entropy bcc phase is defined as HEB phase in this research. The lattice constants of HEB phase are estimated by the rule-of-mixture or Vegard’s law^10^,^13^,^40^,^41^:


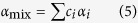


where *c*_*i*_ and *α*_*i*_ are the atomic fraction and the lattice parameter of element *i*. The calculated result of the lattice constants for HEB phase is 3.2306 Å. The experimental results are plotted in Fig. 3(b). With an increase in supercooling from 256 K to 640 K, the lattice constants (*α*) increases from 3.2512 Å to 3.2546 Å, which is expressed by the following equation:


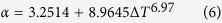


It is clear that the lattice constants derived from XRD analysis results are larger than the calculated value, which indicates that the lattice cells expand obviously due to the rapid solidification under high supercooling. Fig. 3(c~e) show the surface microstructural patterns of high-entropy WMoTaNbZr alloy at the different supercoolings. When the supercooling is small, Δ*T* = 256 K, the surface structure of primary HEB phase is characterized by chrysanthemum structure with the evident branch, the length of HEB phase is about 105 μm. The HEB dendrites are significantly refined with increase of supercooling. For example, its microstructure shows the nubby characteristic with about 80 μm long if the supercooling is 371 K, as seen in Fig. 3(d). However, the dendritic structure disappears once the supercooling exceeds 371 K, which is substituted by the vermicular morphologies without any branches, the length is about 25 μm. Obviously, the supercoolings have the significantly influence on the surface structure of the refractory WMoTaNbZr high-entropy alloy.

The rapid dendritic growth of primary HEB phase always induces a thermal recalescence and interface migration effect, as seen in Fig. 1(a~c), the dendritic growth velocity is measured by the infrared method according to the ratio of sample diameter to recalescence time, the results are illustrated in Fig. 1(d), it increases slowly when the supercooling is less than 355 K. For example, the supercooling Δ*T* is 82 K, the growth velocity *V* is merely 0.23 m/s, if Δ*T* = 355 K, it is only 0.98 m/s. Subsequently, the dendritic growth velocity increases rapidly with the increase in supercooling. Ultimately it achieves 13.5 m/s velocity at the largest supercooling of 640 K. The dendritic growth velocity *V* shows a power law relation to the bulk supercooling Δ*T* and it can be described by the following equation:


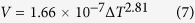


**Figure 4:**
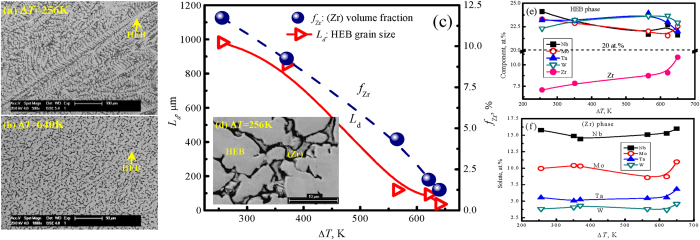
The internal microstructures and phase compositions of rapidly solidified quinary refractory WMoTaNbZr high-entropy alloy versus liquid supercoolings, (**a**) the well-developed HEB phase dendrites in the cross-section of a sample supercooled by 256 K, (**b**) the refined vermicular dendrites of HEB phase within a sample supercooled by 640 K, (**c**) the grain size of primary HEB phase and the volume fraction of minor (Zr) phase, (**d**) the locally enlarged morphologies of major HEB and minor (Zr) phases inside the sample supercooled by 256 K, (**e**) the HEB phase composition varies with liquid alloy supercooling and (**f**) the solute contents of (Zr) phase at different supercoolings.

The microstructures of refractory WMoTaNbZr high-entropy alloy relates to the supercooling and dendritic growth velocity, which are shown in Fig. 4(a,b). For the small supercooling lower than 371 K where the dendritic growth velocity is slow, the primary HEB phase is characterized by the coarse and well-developed dendrites, which displays as the lightgrey phase in Fig. 4(a). Large grains of about 982 μm in length is observed in 256 K, while supercooling of 371 K has a much finer gain size, on the order of 854 μm (shown Fig. 4(c) as the red triangle). Once the supercooling exceeds 371 K, the dendritic feature of primary HEB phase remarkably refines and forms the vermicular structure, the grain size of primary HEB phase is only 36 μm at the largest supercooling of 640 K, which are shown in Fig. 4(b,c). Clearly, the refined microstructures may be induced by the effect of the supercooling, dendrite growth velocity and thermophysical feature together.

A small volume fraction of (Zr) solid solute phase is detected within the solidified samples, which can be seen from Fig. 4(d). Its volume fraction is less than 5%, which is shown in Fig. 4(c). It achieves the largest value at the 256 K, *f*_*(Zr)*_ = 11.73%, afterwards it decreases with the supercooling. Ultimately it has the minimum value, *f*_*(Zr)*_ = 1.25% at Δ*T* = 640 K. Therefore, the refined microstructure could promote the solubility of primary HEB phase and reduces the (Zr) solid solute phase to form at large supercooling.

## Actual solute redistribution characteristics

Energy dispersive spectrometer (EDS) was used to analyze the concentration change of HEB phase and solute redistribution characteristics of solid solute (Zr) phase at different supercoolings, as illustrated in Fig. 4(e,f). In the HEB phase, there are enriched with W, Mo, Ta, and Nb elements, and depleted with Zr element. From Fig. 4(e), the maximum concentration of W, Mo, Ta, and Nb in HEB phase are all larger than the initial concentration of 20 at.%. Despite the obvious variation in the different supercooling, the ultimate concentration is about 22.5 at.%. However, the solute Zr content in HEB phase is far below the original component of high-entropy WMoTaNbZr alloy. At small supercooling, Δ*T* = 256 K, the Zr solubility in HEB phase is 7.07 at.%. Then the obvious increase tendency with the increase in supercooling. At the largest supercooling of 640 K, it attains 10.88 at.%. Therefore, the obvious solute tripping effect occurs under high supercooling and the solute Zr is difficult to dissolve in HEB phase.

The actual solute distribution of (Zr) phase is demonstrated in Fig. 4(f). The solute contents of other components in (Zr) phase have the order of Nb > Mo > Ta > W. It also shows the similar variation tendency for different solutes in (Zr) phase at the different supercoolings. When the supercooling is less than 621 K, the solute contents have the slight decrease tendency. For example, when supercooling increases from 256 K to 621 K, the Nb content decreases from 15.77 to 15.27 at.%, Mo content from 9.96 to 8.72 at.%, Ta content from 5.55 to 5.54 at.%, and W content from 3.83 to 3.7 at.%. However, the solute contents rapidly increase when the supercooling attains the maximum supercooling of 640 K, the solute content displays a rapid increase in (Zr) phase, the Nb content is 15.99 at.%, the Mo content is 10.95 at.%, the Ta content is 6.82 at.%, the W content is 4.6 at.%, respectively. It is clear that the Nb element has a stronger affinity with the Zr element, however the Ta and W elements have the lower affinity with the Zr element.

## The Vickers hardness of HEB phase

**Figure 5:**
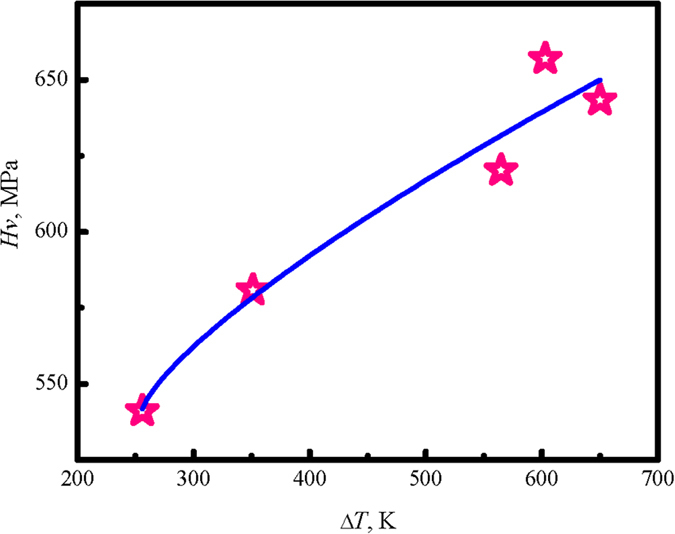
The measured Vickers hardness of HEB phase at different supercoolings.

The mechanical properties are highly related with the supercooling behavior and solidification process. The microhardness of solidified samples have been measured under ESL condition, which shows an increasing tendency with supercoiling for the measured Vickers hardness of HEB phase, as plotted in Fig. 5. When the supercooling is small, Δ*T* = 256 K, the Vickers hardness of HEB phase is 541 MPa. At the largest supercooling of 640 K, it achieves 643.3 MPa Vickers hardness. Apparently, the larger supercooling is, the higher Vickers hardness can be obtained. According to Figs 4 and 5, it indicates that the increase in Vickers hardness is attributed from the grain refinement affect, which originally depends on the supercooling level. Therefore, the supercooling control turns out to be an effective approach to improve the mechanical properties of HEA alloys.

According to above analysis, there should be three reasons for the increase of the lattice constant, dendritic growth velocity and Vickers hardness in refractory WMoTaNbZr HEAs under rapid solidification of highly supercooled. Firstly, the density and the specific heat to emissivity ratio increase with the increase of supercooling, which are easily to result in the lattice expansion and improvement of the Vickers hardness. Secondly, the microstructure refines remarkably that induced by the effect of the significant recalescence, it prompts a large number of Zr atomic diffuse into HEB phase atomic cluster and leads to the increase of lattice constant and Vickers hardness. Thirdly, there is a similar solute content of W, Mo, Ta and Nb elements in the HEB phase, the dendritic growth velocity may be affected by solute diffusion-controlled growth when HEB phase grows in the dendrite ways. Meanwhile, the Zr solute is further suppressed since it has the larger potential barrier during the rapid solidification. Once the supercooling exceeds 371 K, the microstructure refines and the volume of interdendrite (Zr) phase decreases, hence the potential barrier of (Zr) phase weakens tremendously in the process of rapid solidification, the dendritic growth velocity increases rapidly.

## Conclusion

In conclusion, a new quinary refractory WMoTaNbZr high-entropy alloy was successfully synthesized by containerless rapid solidification under electrostatic levitation condition. Its liquidus temperature was determined as 2686 K and it showed a strong liquid state supercoolability to a great extent of 640 K (0.24 *T*_*L*_). The measured liquid density and the specific heat to emissivity ratio both increased linearly with the enhancement of alloy supercooling. The phase constitution was characterized by a major high-entropy body-centered cubic (HEB) phase plus a negligible solid solution (Zr) phase. The dendritic growth of HEB phase always governed the crystallization process and attained a maximum growth velocity of 13.5 m/s at 640 K supercooling. A power function relation between dendrite growth velocity and alloy supercooling was established by the statistical analysis of experimental results. Meanwhile, the Johnson-Mehl- Avrami type transition kinetics was revealed for the transient thermal recalescence process by high speed CCD videography. The microstructures of primary HEB phase transformed from the coarse well-developed columnar dendrites into conspicuously refined vermicular branched structures with the increase of supercooling. The rapid dendritic growth of primary HEB phase resulted in an apparent solute trapping effect of the segregative Zr component and an evident increasing trend of its lattice constant. The minor solid solution (Zr) phase occupied only 3.7 vol% at most and diminished gradually at large supercoolings. The Vickers hardness of HEB phase shows the increasing tendency with the supercooling.

## Additional Information

**How to cite this article**: Wang, W. L. *et al*. Liquid Supercoolability and Synthesis Kinetics of Quinary Refractory High-entropy Alloy. *Sci. Rep.* **6**, 37191; doi: 10.1038/srep37191 (2016).

**Publisher’s note**: Springer Nature remains neutral with regard to jurisdictional claims in published maps and institutional affiliations.
